# Primary chondrosarcomas of the larynx treated with proton radiotherapy: A single institutional experience

**DOI:** 10.1002/cnr2.1621

**Published:** 2022-05-10

**Authors:** Sean S. Mahase, Bhuvanesh Singh, Richard J. Wong, Ian Ganly, Jay O. Boyle, Snehal G. Patel, Nancy Y. Lee

**Affiliations:** ^1^ Department of Radiation Oncology Memorial Sloan Kettering Cancer Center New York New York USA; ^2^ Department of Radiation Oncology Penn State Cancer Institute Hershey Pennsylvania USA; ^3^ Head and Neck Service, Department of Surgery Memorial Sloan Kettering Cancer Center New York New York USA

**Keywords:** laryngeal chondrosarcoma, laryngectomy, proton therapy, radiation therapy

## Abstract

**Background:**

Primary laryngeal chondrosarcomas are rare entities whose excellent survival rates following resection promote conservative surgical approaches to maintain quality of life without compromising outcomes. There are excellent outcomes in skull base chondrosarcomas treated with maximal safe resection and post‐operative proton therapy. Extrapolating from these findings, we report our institutional experience treating symptomatic or growing laryngeal chondrosarcomas using proton beam therapy.

**Cases:**

Demographic information, clinical characteristics, treatment details, and follow‐up data were collected and summarized. Patients were monitored with serial imaging and examination. Stable disease was defined as no progression of disease on imaging. Two patients underwent subtotal resections followed by post‐operative radiotherapy, while two patients received definitive radiotherapy. All patients are currently alive with stable disease at their last follow‐up.

**Conclusion:**

This case series provides initial evidence for excellent outcomes with maximal safe surgical resection followed by proton beam therapy for patients with symptomatic or growing laryngeal chondrosarcomas. Larger studies are warranted to determine the optimal therapeutic approach.

## 1 INTRODUCTION

Chondrosarcomas of the larynx comprise 0.5–1.0% of all primary laryngeal neoplasms.^1^ Given their rarity, current work‐up and management algorithms are largely based on institutional case series.^1^, ^2^ While their etiology is unclear, laryngeal chondrosarcomas (LC) are thought to originate from uncontrolled laryngeal cartilage ossification, and commonly arise from hyaline cricoid cartilage.^2^, ^3^ While the majority are low‐grade, well differentiated and non‐aggressive, portending a favorable prognosis, histologically differentiating LCs from benign chondromas is difficult.^4^ Distinguishing chondromas and LCs via diagnostic imaging is also challenging: LCs appear as smooth, lobular, well‐circumscribed, hypointense masses with calcifications and associated cartilaginous destruction on computerized tomography (CT) scans.^5^, ^6^ They are predominantly diagnosed in men aged 60–65 who present with hoarseness, dyspnea, dysphagia or a painless neck mass secondary to tumor growth and compression of adjacent structures.^1^, ^2^, ^3^

Observation is a reasonable approach for indolent, asymptomatic LCs. For symptomatic or growing LCs, surgery is the standard of care, with options including total laryngectomy, hemilaryngectomy, CO_2_ laser resection and debulking. Given the excellent long‐term prognosis, with 10‐year survival rates of 95%,^1^, ^2^, ^7^, ^8^ larynx preservation is imperative to conserving vocal function and optimizing quality of life. Accordingly, most studies focus on surgical considerations and outcomes. Radiotherapy is inconsistently employed for recurrent disease following resection or positive margins, but it's effectiveness for these indications is controversial.^1^, ^2^, ^7^, ^8^, ^9^ Improved image guidance and treatment planning innovations culminated in more precise radiotherapy delivery. These technological improvements are further advanced using proton therapy, facilitating more conformal treatment than photon beam therapy, promoting high integral dose delivery to the targeted lesion while minimizing side effects, and preserving post‐surgical laryngeal function.^10^ While there is a dearth of data evaluating modern radiation therapy techniques in LCs, the skull base chondrosarcoma literature suggests maximally safe surgical resection followed by post‐postoperative proton therapy provides excellent local control and functional outcomes with acceptable toxicity.^11^, ^12^, ^13^ Extrapolating from these experiences, the intention of this study is to provide a modern institutional experience treating LCs utilizing proton therapy.

## 2 MATERIALS AND METHODS

This study was an institutional review board approved retrospective review (16‐1648) of patients with primary LCs who were treated with proton therapy at our proton facility (Procure, Somerset, New Jersey). All studies involving human participants were in accordance with the ethical standards of the institutional research committee and with the 1964 Helsinki Declaration and its later amendments or comparable ethical standards. Electronic medical records and all available dosimetric data for eligible patients were reviewed.

### 2.1 Radiation therapy

All patients underwent CT simulation in the supine position with 3‐point or 5‐point head and neck masks. Positron emission tomography/CT and magnetic resonance (MR) simulation were co‐registered to the CT simulation to facilitate target volume delineation. Gross tumor volume (GTV) was defined as any gross primary detected clinically or radiographically following resection. A clinical target volume (CTV) was created from an isometric 1 cm GTV expansion to account for subclinical disease. A planning target volume (PTV_50) was generated by adding 3–5 mm margin to the CTV to account for interfraction and intrafraction motion errors. The PTV was used as a volume for plan evaluation of dose coverage. Patients received 50 Cobalt‐Gray Equivalents (CGE) to PTV_50, with a sequential boost to the GTV with a 3–5 mm PTV expansion (PTV_70) to 70 CGE. Elective nodal volumes were not targeted given the historically low rates of nodal involvement, as well as low rates of nodal dissection performed on LCs.^1^, ^3^, ^4^, ^7^, ^8^, ^9^, ^14^ All patients received conventional fractionation defined as 2 CGE per fraction, once daily, 5 days a week.

### 2.2 Proton techniques

All patients were treated with proton therapy using the Proteus 235 system (Ion Beam Applications, Louvain‐la‐Neuve, Belgium). Patients were treated with either uniform scanning beam (US) using the match field technique or pencil beam scanning (PBS), depending on machine availability. Those treated with US technique had custom‐made apertures and compensators to shape the lateral and distal edges of the target, respectively. The PBS was delivered in 2 ways; single‐field uniform dose, with each beam angle delivering a uniform dose to cover the entire volume, or multifield optimization, utilizing the beams to collectively encompass the volume. Monte Carlo algorithm was used as a PBS calculation model. Setup variations of 3 mm and range uncertainties of 63.5% were accounted for in the planning optimization. A RBE value of 1.1 was used in planning and evaluation. Daily orthogonal x‐ray imaging on a 6 degree‐of‐freedom couch was performed for setup verification. CT scans were performed weekly as QA (quality assurance) to evaluate anatomic changes. If significant changes were noted, a QA plan was calculated using the weekly QA CT to ensure accurate dose measurement to the target and organs at risk. Any unacceptable dose coverage required adaptive radiotherapy planning.

### 2.3 Follow‐up

Patients were evaluated weekly during radiation therapy by a radiation oncologist, and if applicable, by a multidisciplinary team including head and neck surgeons, nurses, and advanced practice providers. Patients were followed clinically and radiographically at approximate intervals of 1–3 months after treatment completion, every 3 months up to 2 years, and every 6–12 months thereafter. In general, MRI of the head and neck was performed 3 months after the end of radiotherapy then every 6–12 months or as clinically indicated.

### 2.4 Response

Patients were monitored with serial MRI scans and flexible laryngoscopies during their routine follow‐up visits. They were also monitored for worsening or new symptoms related to their laryngeal chondrosarcoma, including hoarseness, voice strength, and swallowing. Stable disease following radiotherapy was defined as no progression of disease on imaging and no progressive or new symptoms attributable to their tumor. Progression‐free survival and overall survival (OS) were determined from date of treatment completion to date of last follow‐up.

## 3 CASES

### 3.1 Patient selection and characteristics

We queried our institutional records for all patients with primary LCs treated with proton beam radiotherapy. This cohort entailed four Caucasian males with a mean age of 53 (Table 1). All patients were evaluated by an otolaryngologist and a radiation oncologist with subsequent discussion of their case and treatment options at our multidisciplinary head and neck case conference. Two patients underwent subtotal resection followed by post‐operative radiotherapy, while two patients received definitive radiotherapy (Table 2). All patients were treated with proton therapy to 70 CGE (Table 3).

**TABLE 1:** Individual patient and tumor characteristics

Case number	Age (years)	Gender	Race	KPS	Smoking History	Tumor location	Grade	Maximum dimension (cm)
1	48	M	Caucasian	80	Former 5 pack‐year smoker; quit 10 years ago	Cricoid	1	3.8
2	46	M	Caucasian	90	Non‐smoker	Cricoid	1	2.3
3	52	M	Caucasian	90	Non‐smoker	Cricoid	1	2.7
4	68	M	Caucasian	90	Former 1 pack‐year smoker; quit 50 years ago	Cricoid	2	4.6

Abbreviations: KPS, Karnofsky performance status; M, male.

**TABLE 2:** Treatment details and outcomes

Case number	Treatment	Surgery (if applicable)	Dose (CGE)	Fraction size (CGE)	Acute AE	Chronic AE	Post‐treatment Voice quality	PFS (months)	OS (months)	Salvage (if applicable)	Current status^a^
1	Surgery + radiotherapy	Laser excision of intraluminal component of the mass (STR)	70	2	Moderate odynophagia; moderate dermatitis, hoarseness	None	Improved	10	10	N/A	SD
2	Surgery + radiotherapy	Transoral endoscopic debulking (STR)	70	2	Hoarseness, thyrotoxicosis, mild dermatitis	None	Improved	19	19	N/A	SD
3	Definitive radiotherapy	N/A	70	2	Mild odynophagia; mild dermatitis	None	Improved	60	60	N/A	SD
4	Definitive radiotherapy	N/A	70	2	Mild odynophagia; moderate dermatitis	Moderate odynophagia, tracheoesophageal fistula	NA	5	12	laryngectomy	SD^b^

Abbreviations: CGE, Cobalt–Gray–Equivalent; N/A, note applicable; PFS, progression‐free survival; OS, overall survival; SD, stable disease; STR, subtotal resection.

^a^ Stable disease defined as no progression of symptoms or of lesion on imaging.

^b^ Stable disease after salvage procedure.

**TABLE 3:** Dosimetry of planning target volume and critical organs at risk

	PTV_70					Esophagus			Spinal cord	
Case number	Mean (cGyRBE)	Max (cGyRBE)	D95 (cGyRBE)	V95 (%)	V100 (%)	Mean (cGyRBE)	Max (cGyRBE)	V60 (cc)	D 0.1 cc (cGyRBE)	Surface max (cGyRBE)
1	7147.0	7329.0	6855.0	99.43	97.09	3743.0	6951.0	2.74	2091.0	2605.0
2	7297.0	7888.0	6812.0	97.76	89.36	1020.0	7464.9	2.02	290.0	671.0
3	6285.0	7259.0	6220.0	100.0	97.72	N/A^a^	N/A^a^	N/A^a^	17.0	81.00
4	7158.0	7627.0	6011.0	99.55	99.26	319.0	7018.0	0.10	556.0	1109.0

Abbreviations: PTV, planning target volume; cGyRBE, centi‐Gray relative biological equivalent; cc, cubic centimeter; N/A, not applicable.

^a^ Treated lesion located superior to esophagus; no overlap with PTV.

### 3.2 Case 1

**FIGURE 1:**
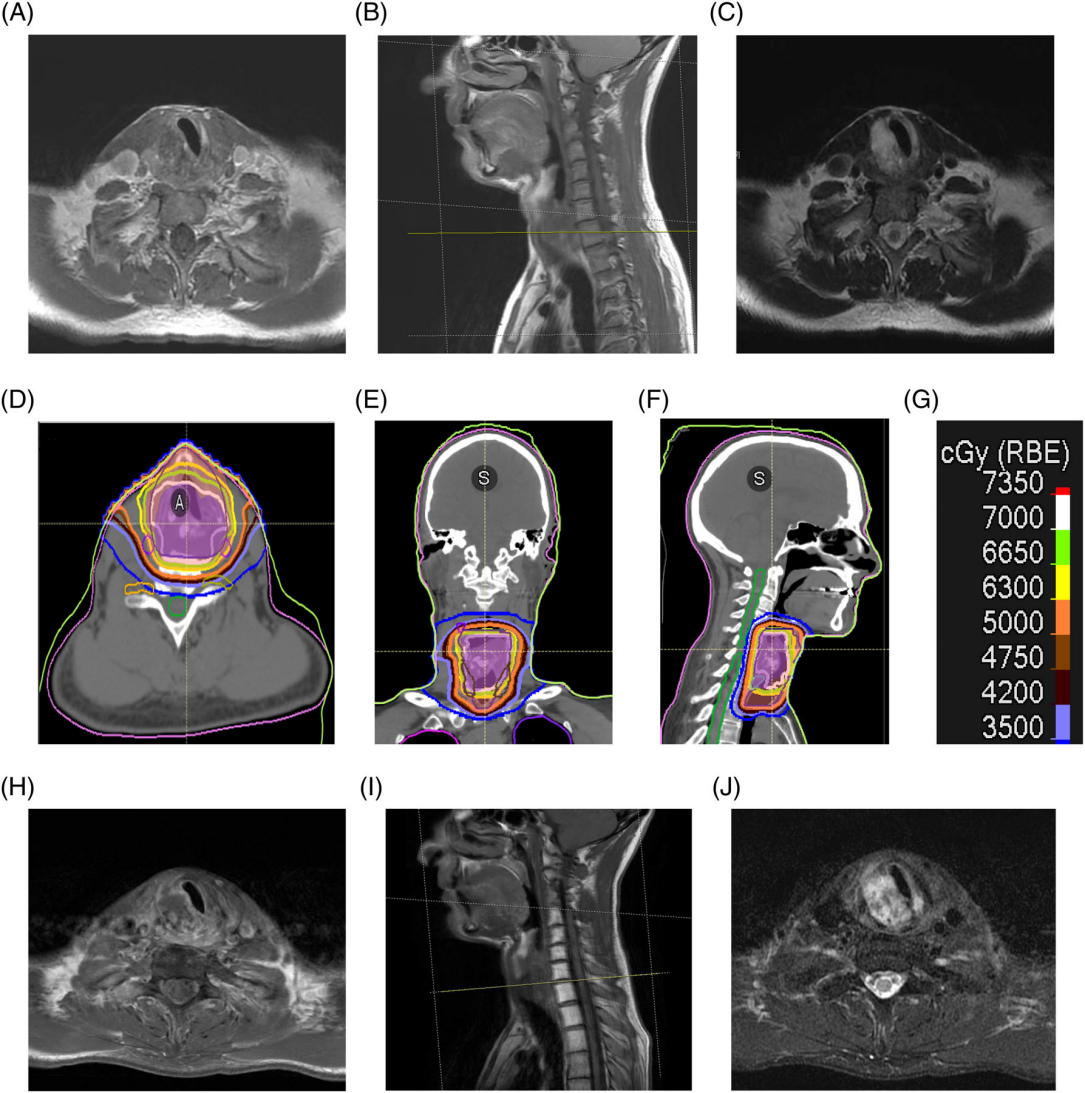
Representative pretreatment (A) axial T1, (B) sagittal T1 and (C) axial T2 imaging of case 1's chondrosarcoma. Dose distribution for radiotherapy plan (D–F) with corresponding isodose line key (G). Representative (H) axial T1, (I) sagittal T1 and (J) axial T2 imaging at last follow‐up

A 48‐year‐old man presented with shortness of breath on exertion of 3 week's duration, and decreased vocal strength with an inability to speak for prolonged periods. He sought evaluation at a local hospital where workup revealed a 2.6 × 3.4 cm right cricoid mass on CT (Figure 1). Ultrasound‐guided fine‐needle aspiration of the mass showed limited cellularity and chondromyxoid material. He presented to our institution for a second opinion. Physical examination was unremarkable. Laryngoscopy showed a submucosal mass in the postcricoid area and medial wall of the pyriform sinus, anteromedial deviation of the right arytenoid cartilage and the vocal cords, and right vocal cord paralysis. MRI demonstrated a 3.4 × 1.8 × 3.2 cm posterior right‐sided cricoid mass with imaging characteristics suggestive of chondrosarcoma associated with right vocal cord paralysis and moderate subglottic tracheal narrowing. There was no adenopathy. After a discussion of treatment options and presenting his case at multidisciplinary tumor board, the patient consented to laser excision of the intraluminal component of the cricoid mass followed by radiotherapy. Pathology from his subtotal resection was consistent with low‐grade chondrosarcoma. He completed radiotherapy 5 months after his laser excision. He tolerated treatment well, experiencing moderate throat pain, hoarseness and moderate dermatitis in the treatment area. All of his radiotherapy‐related side effects completely resolved within 5 months of treatment completion and he noted improvement in his voice quality and strength. He is currently 10 months post‐treatment with stable disease on imaging with no long‐term side effects and preservation of his voice quality.

### 3.3 Case 2

A 46‐year‐old man had an MRI of his cervical spine performed for evaluation of neck pain. This revealed an incidental mass in the posterior cricoid lamina. He was seen by an otolaryngologist at another institution who performed a biopsy of the mass significant for a low‐grade cartilaginous tumor. CT scan showed a 1.4 × 2.3 × 1.8 cm mass in the posterior cricoid lamina protruding into the posterior larynx. Both the anterior and posterior cortices of the cricoid lamina were destroyed. There was no nodal disease. He subsequently underwent transoral endoscopic debulking. His chondrosarcoma measured 2.3 × 0.9 × 1.4 cm 1 month after surgery, indicating a small reduction in size. However, his lesion measured 2.3 × 1.2 × 1.4 cm on CT 6 months after his subtotal resection, concerning for interval growth, prompting a second opinion at our institution. Examination and laryngoscopy were unremarkable. Treatment options discussed entailed total laryngectomy, further debulking and radiation therapy. He completed radiotherapy 13 months after his surgery with all acute symptoms resolving within 3 months of treatment completion. He is currently 19 months post‐treatment with stable disease on imaging, no long‐term side effects, and preservation of his post‐treatment vocal quality and strength.

### 3.4 Case 3

A 52‐year‐old man presented with hoarseness of 1.5 year's duration. Initial evaluations were unremarkable until a right‐sided subglottic mass was identified at his last otolaryngology visit. A CT scan showed 2.7 cm lesion involving less than half of the cricoid cartilage underneath the arytenoid cartilage on the right side. He underwent biopsy which was complicated by life‐threatening hemorrhage, and was taken back to the operating room for cautery. He continued to have intermittent bleeding for 2 weeks. Pathology was consisted with a low‐grade chondrosarcoma. He subsequently established care with our institution. Following multidisciplinary discussion of treatment options, he opted for definitive radiation to minimize treatment‐related morbidity. He developed mild odynophagia and mild dermatitis during radiotherapy which resolved within 6 months of completing treatment. He is currently 5 years post‐treatment with stable disease on imaging, no long‐term side effects, and preservation of his post‐treatment vocal quality and strength.

### 3.5 Case 4

**FIGURE 2:**
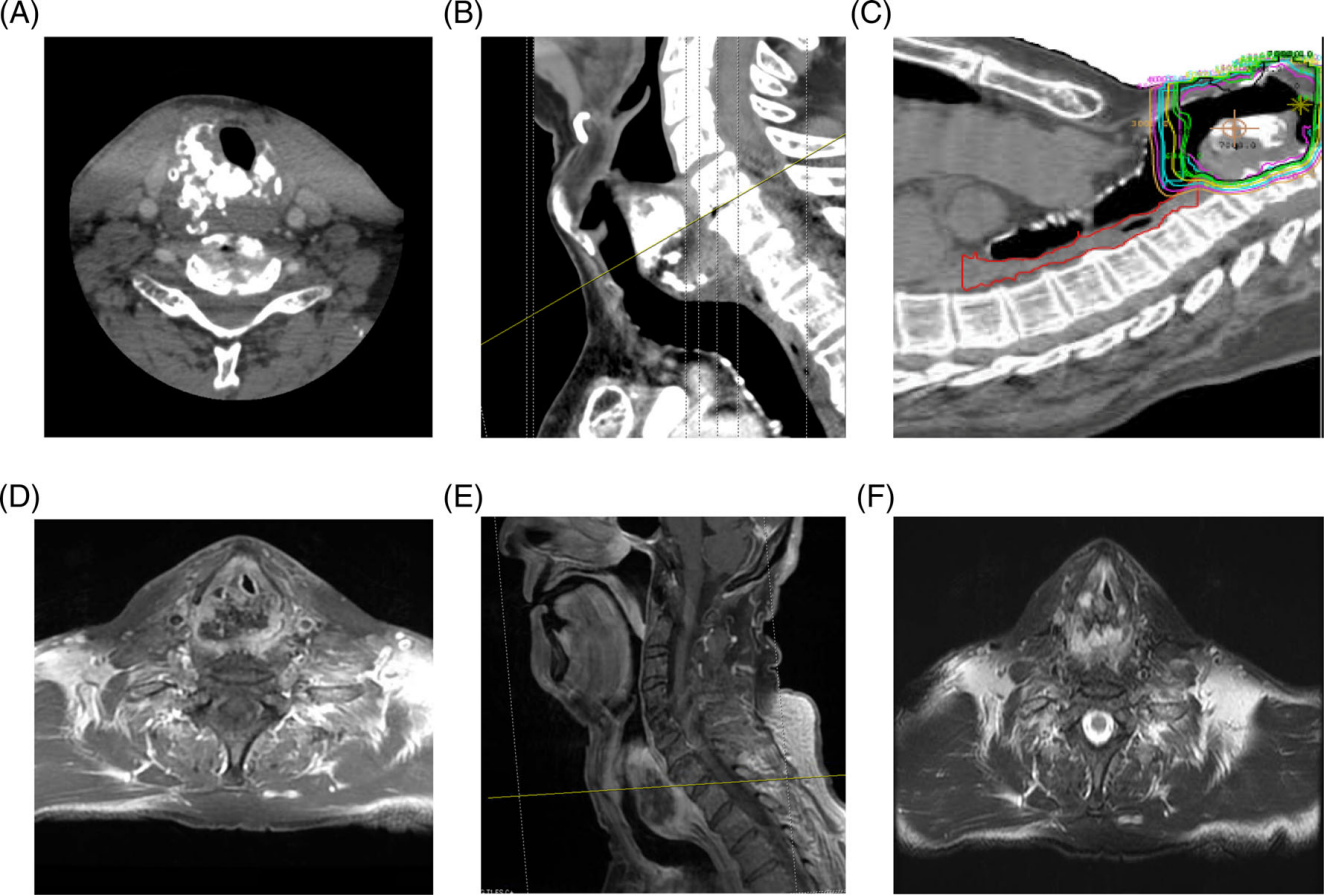
Representative (A) axial and (B) sagittal CT images of case 4's chondrosarcoma at initial presentation. Dose distribution for radiotherapy plan (C). Isodose lines (cGy): red = 7600; black = 7000; green (inner) = 6860; green (outer) = 6650; yellow = 6000; teal = 5000; purple = 4000; orange = 3000. Representative (D) axial, (E) sagittal T1‐weighted and (F) axial T2‐weighted post‐contrast MRI images demonstrating chondrosarcoma 5 months after completion of radiotherapy

A 68‐year‐old man initially presented to an outside institution with hoarseness and was initially diagnosed with gastroesophageal reflux disease. His course was uneventful until his hoarseness acutely worsened 5 years later with associated dysphagia to dry foods and globus sensation. Ultrasound demonstrated a neck mass, prompting a neck CT revealing a complex calcified mass beginning at the level of the cricoid cartilage with extension into the posterior right lateral aspect of the trachea measuring 4.6 × 3.9 cm (Figure 2). No lymphadenopathy was identified.

He was evaluated by an otolaryngologist at our institution. Laryngoscopy was remarkable for a diffuse submucosal bulge in the post cricoid region with no overlying mucosal abnormality. The right piriform sinus was asymmetrically more dilated, and the right true vocal fold was less mobile compared to the left. Biopsy demonstrated a low cellularity cartilaginous neoplasm. However, in the context of his imaging, there was a high index of suspicion for a low‐grade chondrosarcoma, despite the low cellularity and lack of cytologic atypia seen in the limited material. Management options discussed included total laryngectomy, as larynx preserving options were not feasible given the extent of cricoid cartilage involvement, and observation. He opted for surveillance with stable symptoms and no change in disease routine imaging until a CT larynx 2 years later demonstrated approximately 5 mm growth in the lesion in comparison to initial imaging.

After discussion of surgical, systemic and radiotherapy options, he opted for definitive radiotherapy. He received 70 CGE in 35 fractions and tolerated the treatment well with only mild odynophagia and moderate erythema of the neck. Due to geography and the COVID pandemic, following treatment completion he was managed by his local primary physician and otolaryngologist while maintaining regular contact via telehealth. Three months after completing treatment he experienced persistent odynophagia but had improved voice quality. Laryngoscopy showed no focal abnormalities related to radiotherapy. An MRI performed 5 months after completing radiotherapy showed the tumor measuring 4.9 × 4.1 cm on axial plane; previously 4.7 × 3.8 cm pretreatment. Prominent enhancement and signal abnormality within the adjacent endolaryngeal and paralaryngeal soft tissues were most consistent with evolving radiation changes. Six months post‐radiotherapy, he continued to experience persistent pain rated 5/10 in the irradiated treatment area. This was managed with various medications, including ibuprofen, gabapentin, tramadol and oxycodone. He lost 22 pounds since start of treatment. At 7 months post‐treatment, he presented to his local hospital with acute difficulty swallowing liquid and was diagnosed with a tracheoesophageal fistula. A PEG tube was placed at this time. At 9 months post‐treatment he presented with shortness of breath of 4 day's duration. There was no change in his fistula and he was completely dependent on his PEG for nutrition. To protect his airway protection and preservation long‐term swallowing he underwent salvage laryngectomy, right partial pharyngectomy with pectoralis flap, and myocutaneous reconstruction of the pharyngeal defect. Pathology demonstrated a grade 2 chondrosarcoma, 4 cm in greatest dimension, involving cricoid and extending into the right pharynx with negative margins. He is doing well overall since his surgery with no evidence of disease recurrence or new symptoms.

## 4 DISCUSSION

Primary chondrosarcomas of the larynx are rare tumors whose management is largely based on institutional case series. Based on the excellent outcomes derived from these studies, observation is an acceptable option for indolent, asymptomatic LCs. Maximal safe resection is recommended for symptomatic tumors, or growing LCs at risk of becoming symptomatic to confer excellent survival rates while preserving vocal function and maintaining quality of life.^1^, ^2^, ^7^ While LCs can exhibit locally aggressive behavior, database analyses demonstrate regional nodal positivity rates of 0.6–1.2%,^8^, ^14^ thus surgical management entailing partial or total laryngectomy, or laser excision, do not include nodal dissection in the absence of clinically suspicious lymph nodes.^1^, ^3^, ^4^, ^7^, ^8^, ^9^Thompson et al., reported one of the largest LC case series entailing 111 cases primarily treated surgically between 1970 and 1997. There was a 96.3% survival rate with a mean 10.9 years follow‐up. Only five patients received post‐operative radiotherapy: one patient was grade 1 while the other four were grade 2; two underwent wide excision, two had laryngectomies and one had a partial laryngectomy. Four patients were alive at last follow‐up while the fifth patient died of metastatic disease. Further radiotherapy details were not reported.^1^

Recently, a systemic review and two national database studies provided analyses on larger patient cohorts. Chin et al. complied published literature on LCs, comprising 513 patients with an average tumor size of 3.7 cm. Local excision, total laryngectomy, partial laryngectomy, laser excision and endoscopic excision were performed on 30.1%, 29.4%, 15.0%, 3.9%, and 3.4% of patients, respectively. Radiotherapy was used as a primary treatment modality in five patients and as adjuvant treatment in 16 cases. The rationale for utilizing radiotherapy in these cases, as well as treatment details, were not discussed. Disease‐specific survival rates at 1, 5, 10, and 20 years were 97.7%, 91.4%, 81.8%, and 68.0%, respectively.^7^ A national cancer database (NCDB) study by Talati et al. queried all LC cases from 2004 to 2016. Among the 348 patients included, most LCs were low grade (grade 1: 46.0%; grade 2: 35.1%), and mean tumor size was 3.87 cm. Primary surgical intervention was performed in 81.6% of cases, of which 37.0% underwent a partial laryngectomy and 32.7% underwent a total laryngectomy. Only 6.6% of the cohort received adjuvant radiotherapy, and there was no mention of radiotherapy used as a definitive modality. Tumors with positive margins underwent radiation therapy at a similar rate to tumors with negative margins (3.9% vs. 8.1%). On multivariate analysis, total laryngectomy did not provide a survival benefit over partial laryngectomy, or local excision, and adjuvant radiation did not improve survival compared to surgery alone. These patients had a >95% OS at 1 year and mean survival of >10 years.^8^ A second NCDB including 274 cases between 2004 and 2016 reported similar results regarding demographical data, treatment approaches and outcomes. Adjuvant radiotherapy was utilized in 5.5% of cases, and definitive radiotherapy was used in only 1.8% of cases.^14^

There is a dearth of data evaluating the role of radiotherapy in LCs. Gripp et al. published a literature review of all LCs treated with radiotherapy up to 1985. Their review found only eight evaluable cases: four had total laryngectomy, two had partial laryngectomy, one had debulking and one only underwent a biopsy. In these cases, radiotherapy was used for residual tumor, local relapse, and unspecified reasons.^15^ They discussed the difficulty assessing response to radiotherapy, as durable local control can be obtained even if there is no change in tumor extent,^15^ which has also been considered in other reports,^16^, ^17^, ^18^ and may contribute to the notion that these are radioresistant tumors. Thompson et al. noted that patients diagnosed before 1980 had a significantly worse prognosis than those diagnosed afterward, owing to the increasing ubiquity of CT scans enabling better pre‐operative evaluation of tumor size and extent to guide surgical decisions. Additionally, surgical techniques improved with the introduction of microscopic and laser techniques.^1^ Radiotherapy has seen similar advancements with improved image guided treatment delivery and planning algorithms, as well as utilization of proton beam therapy,^10^ thus warranting an update on outcomes with this modality. However, recent publications entailing radiotherapy in LCs are limited to case reports of single patients^19^; none utilizing proton therapy.

Proton beam therapy has minimal exit dose after depositing its' energy (Bragg peak), promoting more conformal treatment than photon beam therapy, thus minimizing radiotherapy dose to surrounding healthy structures and enabling safer dose escalation to the treatment target.^10^ Skull base chondrosarcomas frequently involve critical intracranial structures, precluding safe gross total resections. Most are treated with a combination of maximally safe resection followed by proton therapy with excellent outcomes.^11^, ^12^, ^13^ The largest study is a single institutional retrospective series of 200 skull base chondrosarcomas treated at Massachusetts General Hospital. All patients underwent varying degrees of surgery: 74%, 21%, and 5% underwent subtotal resection, partial resection or biopsy and gross total resection, respectively. Almost two thirds (64%) underwent one surgery prior to radiotherapy, whereas the remainder had up to six operations for either tumor progression or as part of a staged procedure to improve proton therapy targeting. All patients subsequently underwent fractionated proton therapy with doses ranging from 64.2 to 79.6 CGE (median, 72.1 CGE in 38 fractions). Tumor control was defined as lack of progression by clinical and radiographic assessment. The 5‐ and 10‐year local control rates were 99% and 98% respectively, and the 5‐and 10‐year disease‐specific survival rates were both 99%.^12^ A systematic review of skull base chondrosarcomas treated with maximally safe surgical resection and post‐operative proton therapy between 1980 and 2008 also demonstrated high long‐term local control and survival using doses >60 Gray with an acceptable toxicity profile.^13^

The aforementioned studies promote conservative approaches entailing larynx‐preserving, maximal safe resection to preserve quality of life in patients with symptomatic or growing LCs.

Given the relatively slow growth of these tumors, frequent follow‐up would allow adequate time to perform a repeat surgery for recurrent disease. The added benefit of radiotherapy is difficult to determine outside of a study designed to assessed local control, and preservation of quality of life and vocal function given the excellent survival rates cited above in parallel with few cases receiving adjuvant radiotherapy. An alternative is a hybrid approach extrapolating from the skull base chondrosarcoma literature entailing maximally safe resection followed by adjuvant radiotherapy.^11^, ^12^, ^13^ Accordingly, in our case series we defined stable disease as no growth of the tumor following treatment and no new or progressing symptoms attributable to the tumor, rather than inducing further regression. Given the average patient age at presentation, OS rates following resection, and slow growth at of these tumors, this strategy can achieve excellent long‐term local control and preservation of vocal function, obviating the need for salvage surgery. Initial maximal safe resection allows for immediate symptomatic relief, airway clearance and pathological confirmation. Radiotherapy effectively targets the remaining tumor volume that could not be resected, mitigating tumor growth and preserving post‐surgical vocal function without significant morbidity. Resection also shrinks the target volume, minimizing the risk of radiotherapy‐induced side effects.

Sarcomas are a heterogeneous entity, with soft tissue sarcomas of the extremity and retroperitoneum demonstrating significantly lower local control and survival rates than LCs, reflective of the more aggressive treatment approach, including larger radiotherapy planning volumes, for these entities.^20^, ^21^ The aforementioned studies on LCs do not detail radiotherapy planning, however our delineation of GTV, CTV, and PTV parallel that of the skull‐base chondrosarcoma radiotherapy literature, entailing a 0–1 cm GTV expansion to create a CTV with a subsequent 0–0.5 cm PTV expansion.^11^, ^12^, ^13^ The planning volume expansions and decision to not electively cover regional lymph nodes in LCs follow surgical approaches focusing on local control in a conservative manner without nodal dissection given the low propensity for nodal and distant metastases and excellent survival rates. While our institutional experience is limited by the overall number of patients and heterogeneity of their management, all are alive with stable disease at the time of this publication. Cases 1–3 demonstrate multidisciplinary management of carefully selected patients resulting in minimal morbidity from either surgery or radiotherapy, with preserved improvement in vocal quality and strength. Conversely, case 4, entailing a relatively large tumor that only received radiotherapy, would likely have benefitted from resection to reduce the size of his radiation field. These, outcomes parallel findings in the previously discussed base of skull chondrosarcoma literature. Together, these cases may help guide multidisciplinary teams regarding strategies for a rare tumor whose treatment paradigm can largely impact long‐term quality of life. Larger studies with an emphasis on local control and functional laryngeal preservation are warranted to determine the optimal therapeutic approach to laryngeal chondrosarcomas, as well as the role of proton beam therapy in their management.

## CONFLICT OF INTEREST

The authors have stated explicitly that there are no conflicts of interest in connection with this article.

## PRIOR PRESENTATION

This work has not been presented at any meetings.

## AUTHOR CONTRIBUTIONS

**Sean S. Mahase:** Conceptualization (equal); data curation (equal); investigation (equal); writing – original draft (equal); writing – review and editing (equal). **Bhuvanesh Singh:** Conceptualization (equal); data curation (equal); investigation (equal); supervision (equal); validation (equal); writing – review and editing (equal). **Richard Wong:** Conceptualization (equal); formal analysis (equal); investigation (equal); supervision (equal); writing – review and editing (equal). **Ian Ganly:** Conceptualization (equal); investigation (equal); methodology (equal); writing – review and editing (equal). **Jay O. Boyle:** Conceptualization (equal); investigation (equal); methodology (equal); writing – review and editing (equal). **Snehal G. Patel:** Conceptualization (equal); investigation (equal); methodology (equal); supervision (equal); writing – review and editing (equal). **Nancy Y. Lee:** Conceptualization (equal); data curation (equal); formal analysis (equal); investigation (equal); methodology (equal); project administration (equal); supervision (equal); validation (equal); writing – original draft (equal); writing – review and editing (equal).

## Data Availability

Availability of data and material: The datasets generated during and/or analyzed during the current study are available from the corresponding author on reasonable request.
